# Peripheral CD4CD8 Double Positive T Cells with a Distinct Helper Cytokine Profile Are Increased in Rheumatoid Arthritis

**DOI:** 10.1371/journal.pone.0093293

**Published:** 2014-03-25

**Authors:** Dagmar Quandt, Kathrin Rothe, Roger Scholz, Christoph W. Baerwald, Ulf Wagner

**Affiliations:** Clinics for Gastroenterology and Rheumatology, Division of Rheumatology, University of Leipzig, Leipzig, Saxony, Germany; University Hospital Jena, Germany

## Abstract

Peripheral CD4CD8 double positive (DP) T cells have been reported to play a role in several autoimmune diseases, virus infections and cancer. In rheumatoid arthritis (RA), both CD4 and CD8 single positive (SP) T cells are known to be involved in the pathogenesis, but the role of peripheral CD4CD8 DP T cells has not been investigated in detail. Anti cyclic citrullinated antibodies (ACPA) positive and ACPA negative RA patients, patients with systemic lupus erythematodes (SLE) and age matched healthy donors (HD) were enrolled in the analysis. The frequencies and phenotype of DP T cells in PBMC were investigated. In addition, DP T cells were quantified in biopsies from rheumatoid synovium. After in vitro restimulation, the cytokine production of DP T cells was investigated in cultures of PBMC. CMV specific cytokine secretion as well as proliferation was analyzed following antigen specific restimulation after an appropriate culture duration. DP T cells were found more frequently in RA patients than in healthy controls or patients with SLE. These DP T cells express αβ TCRs, are of a memory phenotype and share features of both CD4 as well as CD8 SP T cells. Importantly, DP T cells were found to also be present in the rheumatoid synovium. Further characterization of DP T cells from RA patients revealed increased production of IL-21 and IL-4, implying a possible role as T helper cells. In addition, DP T cells in RA seem to contribute to the inflammatory process, because they produce significantly more IFNγ than counterparts from HD and are increased in CMV+ RA patients. Given their capacity to produce a variety of cytokines (IL4, IL21 and IFNγ), their association with ACPA positive RA and their presence in the synovium, we suggest an important role of double positive T cells in the pathogenesis of rheumatoid arthritis.

## Materials and Methods

### Patients and Healthy Individuals

A total of 59 RA patients according to the 2010 EULAR/ACR criteria (female: 46, male: 13, mean age 59.4 years, range 34–79 years) were recruited, among them 39 ACPA^+^ and 20 ACPA^−^ patients. 39% of the RA patients were treated with biologicals in combination with conventional standard therapy. Sex and age distribution in ACPA^+^ versus ACPA^−^ patients was similar. In addition, 8 SLE patients (all female, mean age 44.3 years, range 21–54 years) were included. Blood of 36 HD (female: 21, male: 15, mean age of 57.1 years, range 25–71 years) who never had evidence of a chronic inflammatory disorder were recruited as controls. The 4 RA patients undergoing knee surgery (2 male, 2 female) were all ACPA^+^.

### Ethics Statement

Written consents were obtained from all patients and healthy donors. The local ethics committee of the University of Leipzig approved the study.

### Antibodies and Reagents

RPMI 1640 was from Lifetechnologies. X-Vivo15 media was supplied by Lonza. aCD3, aCD4, aCD8 (recognizing the α chain), aCD28, aCD45RO, aCD56, aCCR7, a-IL17, aTCRα24-Jα18 (clone: 6B11), cytokine secretion assays for IFNγ and IL-4, a-fibroblast microbeads and Cytostim were purchased from Miltenyi. Collagenase, Hyaluronidase and DNAse were all from Sigma-Aldrich. aCD45 and aCD38 were from Immunotools. CFDA-SE was purchased from Molecular Probes/Invitrogen. Intra staining Kit, aCD16, aCD8β and aCD3 were from Beckton Dickinson. aCXCR5 was supplied by R&D Systems and aIL21 was from ebioscience. The Beta Mark TCRVβ Repertoire Kit was supplied by Beckman Coulter. The antibodies were used in different conjugates of FITC, PE, PerCp, APC, APC-Vio770 and PE-Cy7.

### PBMC Generation and FACS Analysis *ex vivo*

PBMCs were isolated from EDTA whole blood or buffy coats. Plasma was always discarded from whole blood samples prior to Ficoll-gradient for PBMCs isolation. Subsequently a erythrocyte lysis step with lysis-buffer was applied. Cells were stained with different antibodies and kept on ice throughout the assay. Live Cell analysis (use of PI) with doublet exclusion (LSR II) were performed on a FACS Calibur ™ or a LSR II (both Beckton Dickinson) using Cellquest, FACS DIVA and FlowJo software.

### CMV Specific Cytokine Production and Proliferation

These assays were performed as described recently. [1] In brief, 1×10^6^ PBMC were CFDA-SE labeled and cultured for 7 days (proliferation) or left unlabeled and cultured for 4 hrs (2×10^6^, IFNγ secretion) in the presence of CMV lysate/control lysate (Microbrix Biosystems Inc) of 3 μg/ml in 24-well plates in X-VIVO 15 medium.

### Short Term Culture and Staining for Cytokine Analysis

PBMCs were cultured in X-Vivo 15 supplemented with 1% of each glutamin and penicillin/streptomycin in a density of 5×10^6^ for cytostim (1∶50) or 3×10^6^ for PMA (20 ng/ml and Ionomycin (0.5 μg/ml). Culture time was 4 hrs for both and Monensin (2 μM) was added to the last 3 hrs of PMA/Ionomycin cultures. Cytokines were either detected with cytokine secretion assays (IFN-γ and IL-4) following the manufactures protocol by Miltenyi or by intracellular staining (IL-21 and IL-17) using an intra staining Kit.

### Tissue Digestions and Leucocyte Extraction

Synovial biopsies from RA patients undergoing surgery were obtained and leucocyte isolation was performed as follows. Tissue was cut into pieces and incubated with an enzyme solution (collagenase, hyaluronidase, DNAse in RPMI) for 90 min and 37° under constant rotation. Single cell suspension was obtained using gauze and smooth mechanical disruption of digested tissue. Subsequently cells were sorted for non-fibroblasts using anti-fibroblast microbeads from Miltenyi. Non-fibroblast were used for FACS analysis and CD45 staining was used additionally to other antibodies in order to discriminate non-leucocytes.

### Statistics

Statistical evaluation was performed using Prism version 3.0cx software. Mann-Whitney test, unpaired student’s t-test and correlation analysis with spearmen were applied.

## Introduction

Peripheral CD4CD8 double positive (DP) T cells have first been identified more than 20 years ago. Like their progenitors in the thymus, they express the coreceptors CD4 and CD8 simultaneously, but in contrast to immature double positive thymocytes, they show varying degrees of coreceptor expression, and display a memory phenotype but no markers of recent thymic emigrants [2]–[4]. DP T cells can be found in the blood of healthy individuals where they account for about 1% of all T cells within PBMCs, but are also present in the skin of melanoma patients and in systemic sclerosis [5], [6]. During the cause of severe virus infections such as HIV and Hepatitis, increased frequency of DP T cells have been described, which are Ag-specific and of high effector potential. [2], [7]. DP T cells can provide B cell help both by production of appropriate cytokines and by cell contact due to their T helper like phenotype [5], [6], but they can also acquire killer like capacity [7], [8].

In rheumatoid arthritis, CD4+ Th cells play a pivotal role in the pathogenesis, as indicated by numerous genetic associations of the disease with polymorphisms in T cell related genes as well as by the clinical efficacy of CD28 co-stimulation [10] and CD4 co-receptor blockade [9] [11]. The peripheral CD4+ T cell pool in RA is characterized by several alterations including a paucity of naïve T cells and recent thymic emigrants, an increased memory pool and a global loss of T cell receptor diversity accompanied by large clonal expansions [12], [13].

B cell autoreactivity is also essential in RA pathogenesis, as indicated by autoantibody production and by the clinical efficacy of B cell depletion [14]. The T cell help required for this has been suggested to involve CD40-CD40L interactions [15], but additionally other receptors such as BAFF-R [16] have been identified more recently. The result is the production of disease relevant autoantibodies like anti-citrullinated peptide antibodies (ACPA), anti-RA33, RF and others. ACPA positive RA in particular has been associated with more severe joint destruction [1], frequent extraarticular manifestations and enhanced subclinical artheriosclerosis [17].

A possible role of CD4CD8 DP T cells in the pathogenesis of RA has not been investigated.

Here we report, that CD4CD8 double positive T cells are expanded in the peripheral blood in ACPA^+^ RA and can also be found in the rheumatoid synovium. DP T cells in RA show features of T helper cells by the production of IL-4 and IL-21. Interestingly, CMV^+^ RA patients show increased frequencies of these cells and their number correlates positively with CMV specific IFNγ producers. In line with this, a high number of these cells have lost the CD28 costimulator and DP T cells of RA patients show higher amounts of IFNγ producers than HD counterparts. Our data are the first demonstrating a role for CD4CD8 double positive T cells in RA.

## Results

### ACPA^+^ RA Patients have Increased Frequencies of Peripheral DP T cells

**Figure 1:**
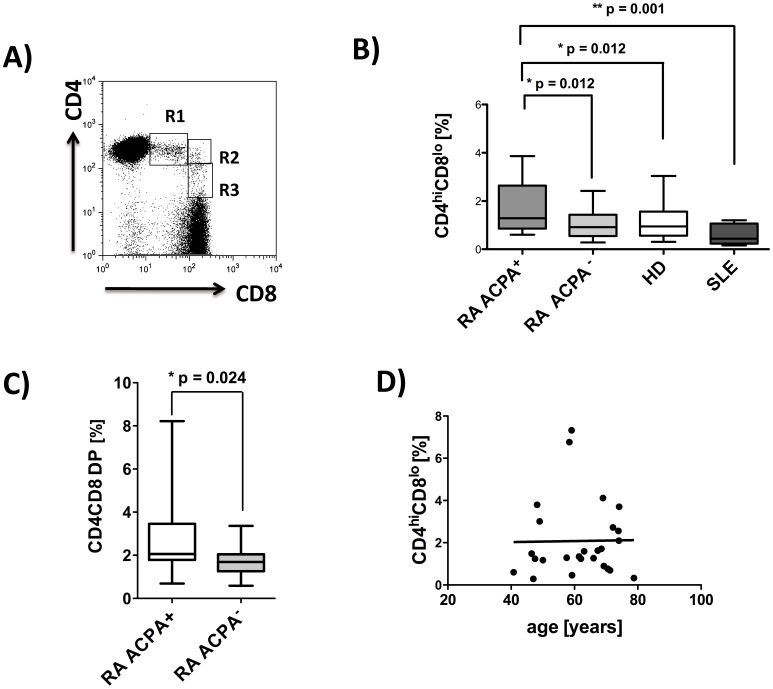
Increased frequencies of peripheral CD4CD8 DP T cells in ACPA^+^ rheumatoid arthritis patients. PBMC were isolated from RA patients (ACPA^+^, n = 37, ACPA^−^, n = 20), healthy donors (n = 36), and patients with SLE (n = 8), and FACS analyses from live cells (PI staining used for exclusion of dead cells) were performed. Analyses for CD4CD8 double positive T cells are always pregated on CD3 positive T cells. **A)** Representative FACS plot from one RA patient, showing CD4CD8 double positive T cells, R1 = CD4^hi^CD8^lo^, R2 = CD4^hi^CD8^hi^ and R3 = CD4^l^°CD8^hi^ **B)** Comparison of frequencies of CD4^hi^CD8^lo^T cells in PBMCs of ACPA^+^/− RA, HD and SLE patients. **C)** Total CD4CD8 DP T cells in ACPA^+^ (n = 19) and ACPA^−^ (n = 20) RA patients. **D)** Correlation analysis for frequencies of CD4^hi^CD8^lo^ T cells with age in ACPA^+^ RA patients. Box plots depict median, interquartile range and 10–90 percentile. Significance as given, *p<0.05, **p<0.01.

Total CD4CD8 DP T cells were quantified as percentage of total CD3^+^ T cells in PBMC, and three subpopulations (CD4^hi^CD8^lo^, CD4^hi^CD8^hi^ and CD4^l^°CD8^hi^) according to the level of coreceptor expression could be distinguished as shown in figure 1A. CD4^hi^CD8^lo^ DP T cells are the most prominent of the DP cell populations and were found to be significantly more frequent in PBMC from ACPA positive RA patients when compared to healthy controls, SLE patients or ACPA negative RA patients (mean%: RA ACPA^+^1.93, RA ACPA^−^ 1.07, HD 1.24 and SLE 0.58, figure 1B). Total frequencies of CD4CD8 DP T cells were also higher in ACPA positive than in ACPA negative patients (figure 1C). The total CD4CD8 DP population will be used in all subsequent displays throughout the manuscript, unless otherwise stated. In the RA patient population no influence of age on the frequency of DP T cells was discernible (figure 1 D). Such a correlation was demonstrated for healthy individuals by others [18] and was also found in our own data for HD. (data not shown) A trend towards a further decrease of DP T cells in SLE patients compared to age matched controls did not reach statistical significance.

### DP T cells in RA Belong to the Memory Pool of TCRαβ T cells and Display No Marker of iNKT cells

**Figure 2:**
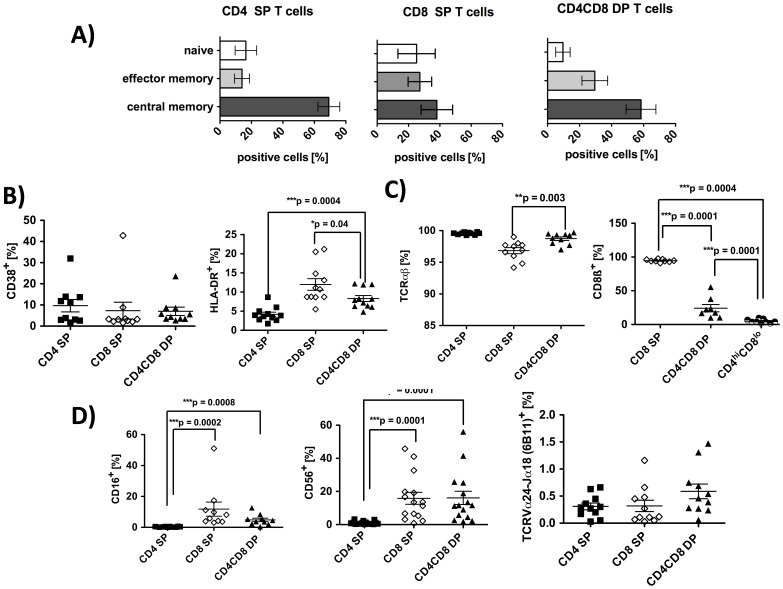
Phenotypic characterization of CD4CD8 DP T cells in RA patients. PBMC from RA patients were isolated and FACS analyses from live cells were performed (PI staining used for exclusion of dead cells). CD3 pregated cells were further gated on CD4 single, CD8 single and on CD4CD8 double positive T cells. **A)** Distribution of CD45RO^−^CCR7^+^ (naive) CD45RO^+^CCR7^−^ (effector memory) and CD45RO^+^CCR7^+^ (central memory) cells in the cell populations indicated. n = 5 5 **B)** Percentage of T cells positive for CD38 (n = 10) and HLA-DR (n = 11). **C)** Percentage of cells bearing a TCR alpha-beta (n = 10) and cells positive for CD8β (n = 9) in the T cell populations indicated. (**D)** Percentage of cells positive for CD16 (n = 10), CD56 (n = 15) and for TCRVα24-Jα18 (n = 11, marker for iNKT) in the T cell populations indicated. In all graphs, lines represent means and SEM. Significance as given, *p<0.05, **p<0.01, ***p<0.001.

The previously described expansion of the memory pool in RA patients [12] was apparent in the analysis of CD4 SP T cells, 70% of which had a central memory phenotype characterized by expression of CD45RO and CCR7 (figure 2A). In agreement with previous reports [2], [7], the majority of CD4CD8 DP T cells was also found to belong to the memory pool (87.9%). Analysis of CD45RO and CCR7 expression identified 58.4% as central memory cells (see figure 2A) and 29.5% as effector memory cells. The described spectrum of memory markers on DP T cells from RA patients did not differ from healthy controls (data not shown).

With regards to activation markers, DP T cells did not differ from CD4 and CD8 SP cells in their expression of CD38. Expression of the T cell activation marker HLA-DR [19], however, was found on DP T cells more frequently than on CD4 SP cells, but less frequently than on CD8^+^ SP lymphocytes (figure 2B). In healthy donors, in contrast, HLA-DR expression on DP T cells did not differ from that of CD4 SP T cells (data not shown. CD56, which can also serve as activation marker for T cells [34], was also found significantly more frequently on DP T cells than on CD4 SP T cells (figure 2D). Other markers associated with activation/regulation like CD69 and CTLA-4 were not expressed on DP T cells (data not shown).

In peripheral blood from RA patients, CD4CD8 DP T cells expressed almost exclusively (99,3%) αβ T cell receptors, which was comparable to CD4 SP T cells (99,3%, figure 2 C). DP T cells were not γ∂ T cells (data not shown). Distribution of αβ and γ∂ TCR expression in the T cell subpopulations was similar in HD (data not shown). In accordance with previous reports [35], CD4^hi^CD8^lo^ DP T cells in RA do not express αβ CD8 heterodimers but αα homodimers, whereas CD8 SP cells are almost exclusively CD8αβ positive (figure 2C).

DP T cells in HD have been described to partially belong to a population of T cells with NK phenotype, called NKT cells. [4] To determine a possible NKT cell phenotype of DP T cells in RA, we used an antibody recognizing the TCRVα24-Jα18:Vβ11, described for invariant iNKT cells, and a combination of anti-CD16 together with anti-CD56 to check for non-invariant NKT cells. The frequency of iNKT cells was not significantly increased in the DP fraction (0.59%±0.13) when compared to CD4 SP or CD8 SP cells (0.31% vs. 0.32%) (figure 2D). Frequencies of CD16^+^ cells were low but were significantly increased in DP T cells (4.8%±1.1) when compared to CD4 SP cells (figure 2D). Co-staining of CD56 and CD16 on DP T cells revealed the presence of some infrequent (2.4%±0.47) CD16^+^CD56^+^ cells which possibly fall into the category of non-invariant NKT cells (data not shown).

**Figure 3:**
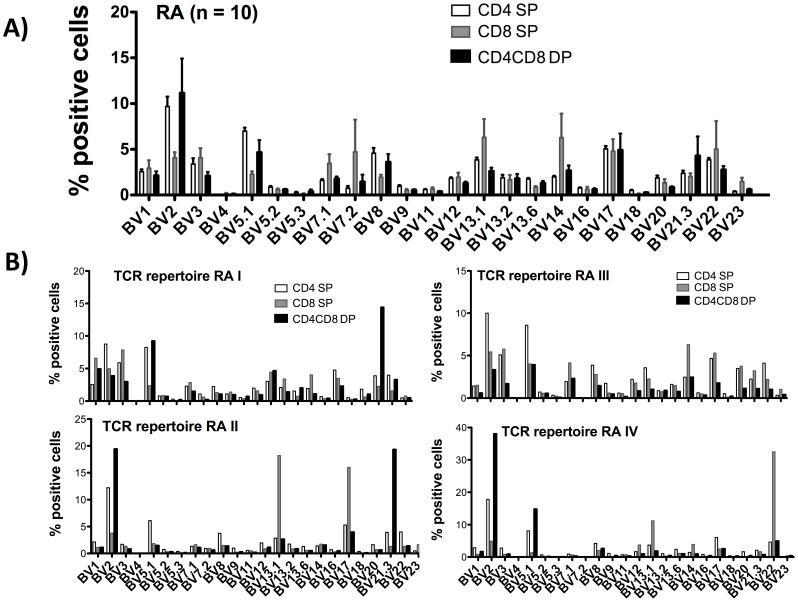
TCR BV distribution in CD4CD8 double positive T cells of RA patients. PBMC from RA patients were isolated and FACS analyses from live cells were performed (PI staining used for exclusion of dead cells). CD3 pre-gated cells were further gated on CD4 single, CD8 single and on CD4CD8 double positive T cells. Bar charts depict the percentage of cells positive for each BV element in the indicated T cell subpopulations investigated. **A)** Mean distribution of 24 TCR BV elements in peripheral blood T cells from 10 RA patients. **B)** Examples of the individual distribution of 24 TCR BV elements in 4 RA patients.

To investigate the TCR repertoire used by DP T cells of RA patients, we performed FACS analysis using 24 different antibodies recognizing a broad range of TCR BV chains. The BV usage within DP T cells of 10 RA patients is divers and does not differ significantly from CD4 SP or CD8 SP. (figure 3A) 5 patients showed a substantial overrepresentation of individual BV elements (BV2, BV17 and/or BV21.3) (representative examples given in figure 3B), possibly indicating antigen-driven clonal expansion.

### DP T cells are Present in the Synovial Tissue and Display Features of T helper Cells

**Figure 4:**
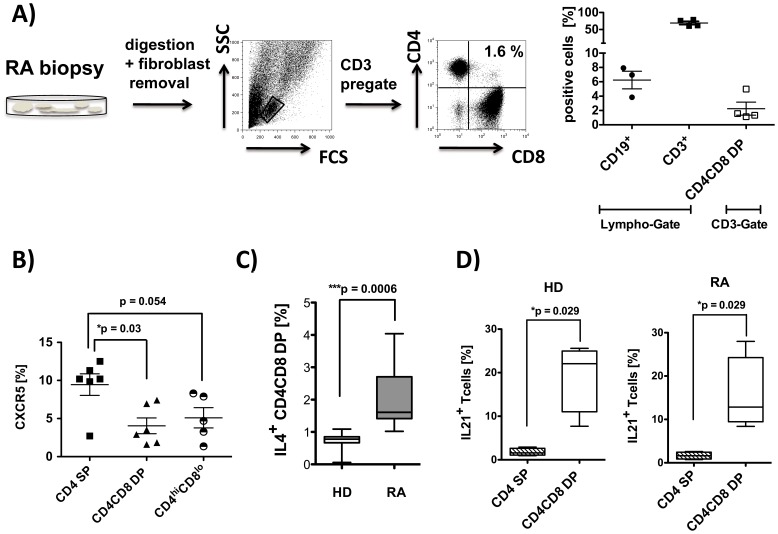
CD4CD8 double positive T cells are present in the synovium of RA patients and produce T helper 2 like cytokines. **A)** Single cell suspensions were prepared from synovial biopsies from RA patients (n = 4) and analyzed by flow cytometry using anti-CD19, anti-CD3, anti-CD4 and anti-CD8 monoclonal antibodies (PI staining used for exclusion of dead cells). Representative FACS plots are depicted. Each data point represents one experiment. **B)** PBMCs from RA patients (n = 6) were isolated and FACS analysis from live cells were performed (PI staining used for exclusion of dead cells). Cells were pre-gated on CD3^+^ T cells, and CXCR5 expression was analyzed in the subpopulation indicated. **C)** PBMC were stimulated in vitro for 4 hrs with Cytostim. Subsequently, an IL-4 specific secretion assay was performed, and expression of CD4 and CD8 was determined in vital cells (PI staining used for exclusion of dead cells) pre-gated on CD3. Percentage of IL-4 producing CD4CD8 double positive T cells in HD (n = 7) and RA patients (n = 8) is given. **D)** PBMCs from HD (n = 4) and RA (n = 4) were stimulated with PMA/Iono for 4 hrs, and intracellular staining for IL-21 was performed. Cells are counterstained with anti-CD3, anti-CD4 and anti-CD8. Depicted is the percentage of cytokine producers in total CD4CD8 DP T cells and in CD4 SP cells. Box plots in C) and D) show median, interquartile range, and 10–90 percentiles. Significance as given, *p<0.05.

The target organ in rheumatoid arthritis is the synovium. Therefore we were interested in identifying DP T cells in the synovium of inflamed joints. As demonstrated in figure 4A we do find CD4CD8 double positive T cells (pregated on CD3^+^ T cells) in the synovium (mean: 2.5%). Of note, the same RA patients also showed CD19^+^ B cells in the synovium (figure 3A).

To explore the potential of CD4CD8 DP T cells to provide B cell help, the CXCR5 expression was analyzed, since this chemokine receptor has been described to be present on several subpopulation of T follicular helper (Tfh) cells, although with varying support for antibody secretion [20]. The results showed that DP T cells from RA patients can express CXCR5, although the fraction of CXCR5 positive cells is higher in CD4 SP than in CD4CD8DP cells (mean: 9.5% vs. 4.1.%, p = 0.03, figure 4B). Similar frequencies were found in DP T cells from healthy individuals (mean 5.1%, data not shown).

As functional and possibly more specific markers of Tfh cells, the ability to secrete IL-4 and IL-21 following restimulation in vitro was determined, since both cytokines are required for B cell help [20], [21]. Determination of IL-4 secretion of DP T cells upon in vitro short term restimulation with Cytostim showed a substantial fraction of them to produce IL-4 (figure 4C). Importantly, this percentage of IL-4 producers was significantly higher in RA patients than in healthy controls (mean: 1.99% vs. 0.71%, p = 0.0006).

Most importantly, however, a large fraction of DP cells (15–19%) was found to produce IL-21, compared to only 2% of CD4 SP T cells (figure 4D). No difference between the IL-21 production of DP T cells from healthy donors compared to those from RA patients was detectable (figure 4D).

### DP T cells in RA Preferentially Produce IFNγ but not IL-17

Several abnormal T cell subpopulations are pathogenetically relevant in RA, among them Th17 cells and IFNγ producers [22]–[24]. Using cytokine secretion assays as well as intracellular FACS analysis, IFNγ and IL17 expression was determined in vitro after short term restimulation with the MHC-TCR crosslinker Cytostim (IFNγ) or with PMA/Ionomycin (IL-17). Frequencies of cytokine producers were calculated as percentage of total CD4CD8 DP T cells, since cytokine expression was not restricted to a particular subpopulation (CD4^hi^CD8^lo^ or CD4^l^°CD8^hi^) of DP T cells.

**Figure 5:**
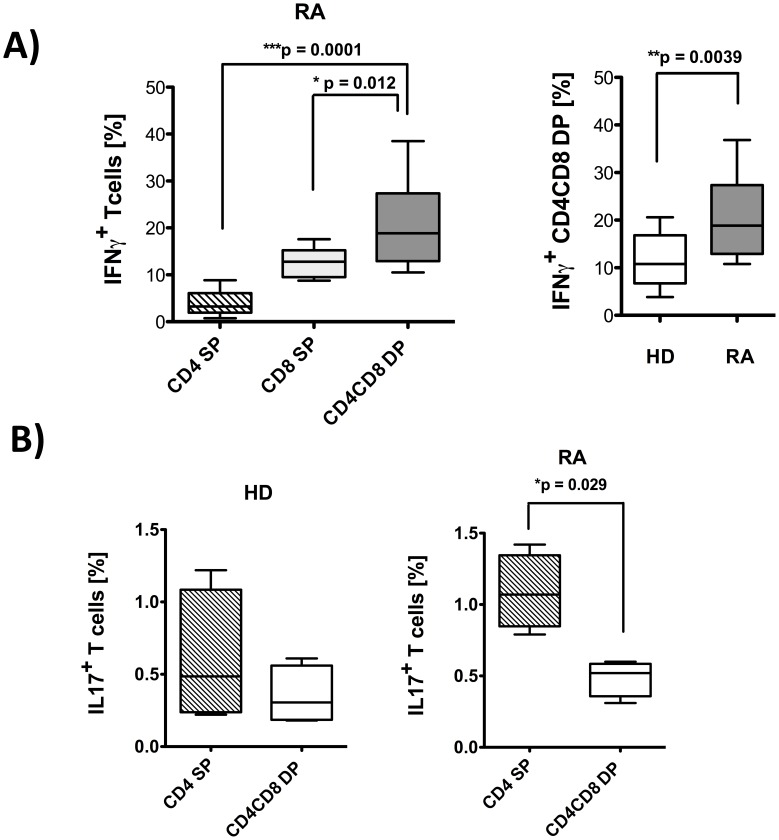
CD4CD8 double positive T cells from RA patients produce inflammatory IFNγ. PBMC from RA and HD were stimulated in(A) or PMA/Iono (B). Subsequently, cytokine specific secretion assays (A) or intracellular stainings (B) were performed and cells were counterstained with anti-CD3, anti-CD4 and anti-CD8. Flow cytometric cytokine analysis were performed on live cells for IFNγ secretion (PI staining used for exclusion of dead cells) or on fixed cells for intra-cellular staining for IL-17, in each case after pre-gating on CD3^+^ T cells. **A)** Percentage of IFNγ producing T cells in samples from rheumatoid arthritis patients (n = 12, left panel and grey box in the right panel) and healthy donors (n = 13, clear box in the right panel) among CD4 SP, CD8 SP and CD4CD8 DP T cells. **B)** Percentage of IL-17 producing CD4 SP and CD4CD8 double positive T cells from RA patients (n = 4) and HD (n = 4). Presented box plots show median, interquartile range, and 10–90 percentiles. Significance as given, *p<0.05, **p<0.01, ***p<0.001.

In RA patients, significantly more DP T cells produced IFNγ than CD4 SP or CD8 SP T cells (mean 21.1% vs. 3.9% or 12.7%, p = 0.0001 and p = 0.012, respectively) (figure 5A). In HD, in contrast, there was no significant difference in the percentage of IFNγ producers between the 3 different T cell populations (CD4 or CD8 SP and DP T cells, data not shown). Most importantly, the frequency of IFNγ producing DP T cells in RA patients significantly exceeds the frequency found in HD (11.4%) (figure 5A).

Results for IL-17 were less conclusive. IL-17 was mainly produced by CD4 SP T cells, and not by CD8 SP or DP positive T cells (figure 5B). Nevertheless, a trend towards higher IL-17 production was seen in CD4 SP T cells from RA patients compared to CD4 SP from HD T cells (figure 5B).

### DP T cells are more Frequent in Latent CMV Infection and Correlate with the Occurrence of CMV Specific Cytokine Producers

**Figure 6:**
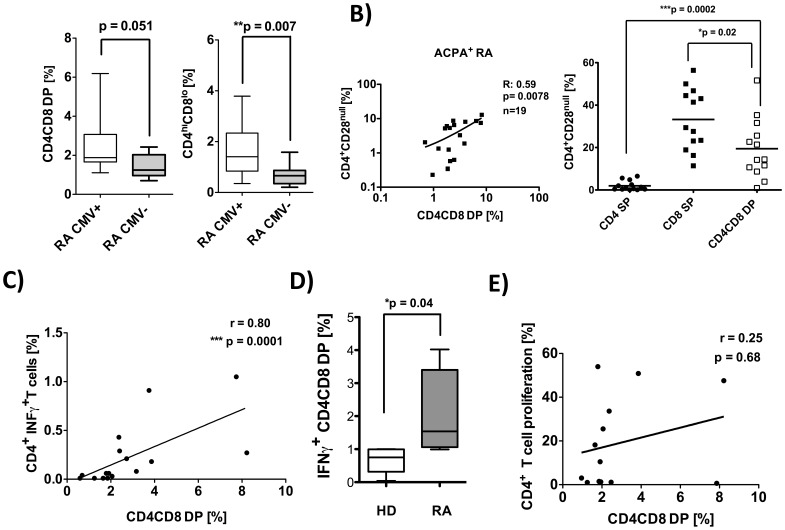
Frequencies of CD4CD8 double positive T cells are increased in CMV positive RA patients and correlate with CMV specific INFγ producers. **A)** PBMC were isolated from CMV^+^ RA patients (n = 28) and CMV^−^ RA patients (n = 12) and analyzed by flow cytometry using anti-CD19, anti-CD3, anti-CD4 and anti-CD8 monoclonal antibodies (PI staining used for exclusion of dead cells). **B)** Correlation of the frequency of total double positive T cells with CD4^+^CD28^null^ T cells in ACPA^+^ RA patients (left graph) and comparison of frequencies of CD28^null^ cells among CD4 SP, CD8 SP and total DP T cells (right graph). **C–D)** PBMCs were restimulated with CMV-lysate for 4 hrs in vitro. Subsequently, an INFγ specific secretion assay was performed and cells were counterstained with aCD3, aCD4 and aCD8. Flow cytometric cytokine analyses were performed on live cells (PI staining used for exclusion of dead cells) pre-gated on CD3^+^ T cells. **C)** Depicted is the **c**orrelation of CMV specific INFγ producing CD4 T cells with the frequencies of CD4CD8 DP cells from the same donor (n = 16). **D)** CMV specific INFγ producing CD4CD8 DP T cells from RA (n = 5) and HD (n = 5) are shown. **E)** PBMCs were labeled with CFDA-SE and restimulated with CMV-lysat (n = 13) for 7 d in vitro. Depicted is the **c**orrelation of CMV specific CD4 T cell proliferation with the frequencies of CD4CD8 DP cells from the same donor. Presented box plots show median, interquartile range, and 10–90 percentiles. Significance as given, *p<0.05, **p<0.01, ***p<0.001.

Latent CMV infection in rheumatoid arthritis is associated with a more severe cause of joint destruction, as published recently by our group and others [1], [17]. This association was limited to ACPA positive RA. Based on the observed increase of DP T cells in ACPA positive RA, which are readily able to produce IFNγ in response to polyclonal stimuli, we decided to stratify the patients according to the presence or absence of anti-CMV IgG antibodies in the serum. The results showed significantly higher frequencies of CD4^hi^CD8^lo^ DP positive T cells, in anti-CMV IgG positive RA patients compared to CMV^−^ patients (mean 1.81% vs 0.71%, p = 0.007, figure 6A). This difference remained significant, if the analysis was restricted to ACPA positive patients only (mean 2.27% vs. 1.55%, p = 0.039). Interestingly, the highest frequencies of CD4CD8 DP T cells (mean%: 2.27) were found in patients simultaneously positive for anti-CMV IgG and ACPA (data not shown).

CD4^+^CD28^null^ T cells are increased in RA patients and associated with a more severe course of the disease [13], [25]. In addition, they are known to occur in the course of a latent CMV infection both in RA patients and in healthy controls. Therefore, the frequency of CD4^+^CD28^null^T cells was determined in the RA patients and found to correlated with the percentages of total CD4CD8 DP T cells (figure 6B). This correlation was restricted to ACPA^+^ RA patients and was not present in ACPA negative patients. Furthermore, DP T cells were found to preferentially have lost CD28 expression (mean%: 19,4, n = 13) in ACPA positive RA patients when compared to CD4 SP (mean%: 1.97) T cells, while the frequency of CD28 negative cells was even higher in CD8 SP (mean%: 33) T cells (figure 6B).

Finally, in order to investigate the CMV response, we restimulated (4 hrs) PBMC from anti-CMV IgG seropositive RA patients with CMV lysate or control lysate and determined the CMV specific IFNγ production of CD4 SP T cells and CD4CD8 DP T cells by cytokine secretion assay. The frequency of CMV specific IFNγ producers was found to correlate with the frequencies of total CD4CD8 DP T cells in CMV^+^ RA patients (figure 6C). Interestingly, the CMV specific IFNγ response of CD4CD8 DP T cells from RA patients was significantly higher (2.09) than in HD (0.67) (figure 6D). The antigen-induced, CMV specific proliferation of CD4 SP T cells, in contrast, did not correlate with the frequency of DP T cells (figure 6E).

## Discussion

The study was conducted to investigate a possible role for peripheral CD4CD8 double positive T cells in the pathogenesis of rheumatoid arthritis.

The results show, that in RA patients, a significant number of mature T cells are indeed double positive for both co-receptors, CD4 and CD8. Those cells, which can also be found in healthy individuals – although in significantly lower numbers – preferentially display a memory phenotype, and have been reported to bear no markers of naïve T cells or recent thymic emigrants [3] which makes it unlikely that they escaped from the thymus as double positive cells.

Instead, their co-receptor expression pattern with higher CD4 than CD8 expression, and the fact that CD4^hi^CD8^lo^ are the most prominent DP T cell subpopulation, as well as the generally presumed pivotal role of CD4+ MHC class II restricted T lymphocytes in RA, suggests that those cells were originally CD4 SP T cells, which gained expression of the second co-receptor CD8. De novo CD8 expression in CD4^+^ T cells under the influence of cytokines like IL-4 has already been described decades ago [26], but the paucity of IL-4 typically found in RA, makes such cytokine driven CD8 expression as an explanation for the emergence of DP T cells in RA less likely. Nevertheless, the increased IL-4 production of DP T cells in RA may sustain their CD8 expression in an autocrine manner.

Two new studies published very recently offer an alternative explanation, since they have identified defined transcription programs which can initiate CD8 gene expression in CD4 SP T cells, for example the silencing of the transcription factor Thpok [27], [28] The precise mechanism underlying the emergence of DP T cells in RA remains to be determined, however.

Increased numbers of double positive T cells were detectable only in ACPA positive and not in ACPA negative RA patients. This suggests their involvement in the autoreactive T cell help provided for auto-reactive, antibody producing B cells in this disease. In line with this, a fraction of the double positive T cells was found to express the typical marker of T follicular helper cells, CXCR5. The percentage of CXCR5 positive cells amongst double-positive T cells was lower, however, than their percentage amongst CD4 SP T cells, which argues against a preferential role of DP T cells as TfH cells. Nevertheless, a proportion of DP T cells might provide B cell help, especially by their production of cytokines with a role in T-B cell collaboration, in particular of IL-21. The site for this B cell help could be non classical follicular centers, which are found in the synovium of a sizable fraction of RA patients. [29], [30].

RA patients with ACPA positive disease have a more severe course of the disease with extra-articular manifestations and increased subclinical arteriosclerosis compared to ACPA- patients [17]. The increased frequency of CD4CD8 DP T cells in our study in ACPA positive RA might reflect the more pronounced immune deviations in those patients and indicates that those cells might be involved in the destructive immune response in this disease.

Phenotypic characterization of DP T cells in RA reveals them to be αβ T cells of a memory phenotype. Similar characteristics have been published for DP T cells in healthy donors [3].

NK T cells have received increasing attention in autoimmunity research recently. DP T cells in RA are not invariant NKT cells, but a small fraction was positive for markers for non-invariant NKT cells. This is in contrast to published data [4] for HD and might indicate a pathogenetically relevant, disease specific role of DP T cell subpopulations in RA. Furthermore, TCR BV analysis reveals an overrepresentation of individual BV elements (BV2, BV17 and/or BV21.3) in DP T cells of some RA patients, possibly indicating antigen-driven clonal expansion.

DP T cells are present in the rheumatoid synovium. In addition, our results show that a high percentage of DP T cells have lost expression of the ubiquitous costimulatory molecule CD28, and that their frequency is increased in CMV^+^ RA patients. CD4^+^CD28^null^ SP T cells are found in 25–70% of RA patients and are associated with a more severe course of the disease [13], [25], [31]. Recently, we reported CMV^+^ RA to be associated with a more severe course of joint destruction [1], and latent CMV infection is known to lead to an expansion of CD4^+^CD28^null^ T cells both in RA patients and in healthy individuals.

The present study showed enhanced frequencies of DP T cells in CMV^+^ RA. Recently, we reported an increased CMV specific IFNγ production in RA compared to CMV^+^ healthy controls. [1] Furthermore, the frequency of DP T cells in the present study was found to correlate with the frequency of CD4^+^ CMV specific IFNγ producers. Interestingly, a low but significant fraction of CD4 SP T cells, and even higher frequencies of the DP T cells in RA do produce CMV specific IFNγ, more frequently though than T cells from HD. This is in line with virus specific DP T cells described in HIV patients [7]. Taken together, those results suggests that DP T cells are at least in part the results of the same RA specific, CMV triggered perturbation in the peripheral T cell pool, which also leads to the occurrence of CD4^+^CD28^null^ T cells. A CMV specific enhanced effector response for DP T cells has already been found in HD [8] and DP T cells are therefore likely also to be involved in the enhanced CMV response in RA.

Independently of CMV specificity, we found DP T cells to produce high amounts of IFNγ, but no IL-17, upon polyclonal stimulation. IFNγ is a key cytokine driving the autoimmune response in RA [23], [32]. DP T cells in healthy, aging individuals are known to be capable of producing IFNγ upon polyclonal restimulation [33]. CD28 negative Th cells in RA have also been shown to secrete IFNγ upon autoreactive stimulation, and in line with this, the DP T cells in RA are also preferentially CD28 negative. Therefore, CD4CD8 DP T cells in RA seem to be part of the pathological T cell pool characterized by replicative exhaustion, aberrant IFNγ production and phenotypic alterations including the loss of the ubiquitous co-stimulatory molecule CD28. DP T cells might be the result of a differentiation program set in place in terminally differentiated memory Th cells, that is switched on under conditions associated with immunosenescence, including the memory inflation observed in latent CMV infection or the chronic autoimmune response in rheumatoid arthritis.

## Conclusion

In summary, we are the first to demonstrate increased frequencies of peripheral CD4CD8 double positive T cells in ACPA positive RA.

DP T cells are present in the inflamed synovium. These cells display features of T helper cells by the production of IL-4 and IL-21. Furthermore, the production of IFNγ, loss of CD28 and the association with CMV positive RA imply a functional contribution to the pathogenesis of the disease.
